# Impact of anthocyanins derived from *Dioscorea alata L.* on growth performance, carcass characteristics, antioxidant capacity, and immune function of Hainan black goats

**DOI:** 10.3389/fvets.2023.1283947

**Published:** 2023-11-21

**Authors:** Haibo Feng, Huiyu Shi, Fengyuan Yang, Yanhong Yun, Xuemei Wang

**Affiliations:** Laboratory of Animal Nutrition and Feed Science, Department of Animal Science, School of Tropical Agriculture and Forestry (School of Agricultural and Rural Affairs, School of Rural Revitalization), Hainan University, Haikou, China

**Keywords:** *Dioscorea alata L.*, antioxidant capacity, immune function, meat quality, Hainan black goats

## Abstract

*Dioscorea alata L.* anthocyanins (DAC) are natural compounds found in plants and have shown potential health benefits. The objective of this investigation was to assess the impact of anthocyanins sourced from *Dioscorea alata L.* on the growth, carcass traits, antioxidant potential, and immune response of Hainan black goats. In this study, 30 three-month-old Hainan black goats (with a weight of 11.30 ± 0.82 kg) were selected and randomly divided into two groups, with 15 goats in each group. During the 60-day experiment, the control group (CON) and the treatment group (DAC) were, respectively, supplemented with 0 and 40 mg/kg BW of DAC in the basal diet. The results showed that DAC had no significant impact on the growth performance and body characteristics of Hainan black goats (*p* > 0.05). However, in terms of meat quality, the addition of DAC significantly increased the pH value and cooking yield 24 h post-slaughter (*p* < 0.05), while reducing the shear force of the meat (*p* < 0.05). Compared to the control group, adding DAC to the feed resulted in a significant increase in the total antioxidant capacity (T-AOC) and superoxide dismutase (T-SOD) concentrations in plasma after 30 days of feeding (*p* < 0.05). After 60 days of feeding, the concentrations of T-AOC, T-SOD, glutathione peroxidase (GSH-Px), and catalase (CAT) in the plasma of the DAC group was higher than that of the control group (*p* < 0.05), while the concentration of malondialdehyde (MDA) was lower than that of the control group (*p* < 0.05). In addition, supplementing DAC significantly increased the content of interleukin-10 (IL-10) and immunoglobulin M (IgM) in the plasma of Hainan black goats after 30 days of feeding (*p* < 0.05), while reducing the content of interleukin-6 (IL-6) (*p* < 0.05). After 60 days of feeding, the immunoglobulin G (IgG) and IL-10 content in the plasma of the DAC group was significantly increased (*p* < 0.05), while the concentrations of IL-1β, IL-6, and tumor necrosis factor-α (TNF-α) were suppressed (*p* < 0.05). In summary, these results indicate that supplementing DAC can improve the meat quality, enhance the antioxidant capacity, and immune function of Hainan black goats.

## 1 Introduction

In ruminant production, various factors such as diet, environment, stress, and the psychological and physiological state of animals can influence the occurrence of oxidative stress in the body (1, 2). Failure to effectively and safely eliminate reactive oxygen species (ROS) can directly or indirectly impair the overall health of ruminant animals, resulting in adverse effects on growth, reproduction, and immune function (3). Oxidative stress is also a significant factor contributing to aging, inflammation, and even diseases. Additionally, meat products are prone to lipid oxidation, resulting in color changes and nutrient loss, which lead to a decline in meat quality (4). Anthocyanins, as natural dietary antioxidants, offer defense against the detrimental effects of oxidative stress (5). Extensive scientific evidence also confirms their diverse range of functions, including anti-inflammatory, immune-regulatory, anti-cancer, and antioxidant properties (6). Furthermore, anthocyanins can prevent the oxidation of milk and meat (7). Previous studies have demonstrated the favorable effects of incorporating anthocyanins as feed additives in animal production, benefiting both animal productivity and health (8). According to previous studies, the consumption of anthocyanin-enriched sugarcane silage has been found to enhance plasma’s total antioxidant capacity (TAC) while also increasing the activity of superoxide dismutase (SOD), catalase (CAT), and glutathione peroxidase (GSH-Px) (9). Additionally, previous studies have shown that composite extracts containing high levels of polyphenols can enhance growth performance and immunity in growing buffalo, while also reducing excretion of methane, nitrogen, and phosphorus (10).

*Dioscorea alata L.*, a yam species with a global presence, is commonly known as the “greater yam” (11) due to its widespread popularity. It is rich in carbohydrates, proteins, fats, fibers, vitamins, minerals, and other components (12) In addition, *Dioscorea alata L*. is one of the important natural sources of anthocyanins, which have antioxidant properties (13). Due to its location in the tropical region, Hainan Island offers favorable climatic conditions for the growth of *Dioscorea alata L*. The extensive cultivation area and high yield contribute to the advantageous extraction of anthocyanins. Previous research has demonstrated the numerous health advantages of dietary supplementation with *Dioscorea alata L.* anthocyanins (DAC). These benefits include improving intestinal damage in mice afflicted with inflammatory bowel disease (14), as well as diminishing oxidative stress in mice (15). Furthermore, DAC effectively mitigates cellular oxidative stress by impeding the phosphorylation of IκB and p65 proteins (16).

To date, there have been limited reports on the application of *Dioscorea alata L*. anthocyanin (DAC) in ruminant animals. Based on our previous investigation, the incorporation of *Dioscorea alata L.* in the diet by replacing 30% of maize revealed significant potential in enhancing the antioxidant capacity of pregnant goats and newborns (17). This finding serves as a crucial foundation for substantiating the viability of this study. The objective of this study was to assess the impact of dietary DAC on the growth performance, carcass characteristics, antioxidant capacity, and immune function of Hainan Black goats. This study holds significant importance in understanding the nutritional value of *Dioscorea alata L.* anthocyanins and their potential applications in animal husbandry. The findings from this research may offer a novel approach for the feeding and improvement of Hainan Black goats, and could potentially provide insights for other livestock industries.

## 2 Materials and methods

### 2.1 Ethics statement

The Ethics Committee of Hainan University (Haikou, China) provided ethical approval for the animal study, under the license number HNUAUCC-2021-00082.

### 2.2 Relative composition of *Dioscorea alata L.* anthocyanins (DAC)

The required *Dioscorea alata L.* for this experiment were provided by the *Dioscorea alata L.* Base at Hainan University, while *Dioscorea alata L.* extract was prepared by Shaanxi Zhongwei Biological Engineering Co., Ltd. The extraction method for DAC was described in detail by Qiu et al. (15). In brief, fresh *Dioscorea alata L.* were extracted using ethanol to form a solution, which was then filtered, concentrated, and spray-dried to obtain DAC. Table 1 presents the abundance of individual anthocyanins, which were identified using a combined LC–MS technique (18, 19), revealing their relative concentrations.

**Table 1:** Anthocyanin profifile of *Dioscorea alata L.* (DAC).

Compound	Rt (min)	MSn (*m*/*z*)	Metabolite name	Relative composition (%)^(a)^
1	9.383	935.24329	Cyanidin3-O-(6’’’-caffeoylsophoroside)-5-O-glucoside	68.4408%
2	10.69	787.20471	Petunidin3-O-(6’’-p-coumaroylglucoside)-5-O-glucoside	7.4824%
3	7	893.23419	Cyanidin3-O-(6’’-p-hydroxybenzoylsophoroside)-5-O-glucoside	5.0421%
4	10.519	303.04956	Delphinidin	4.1572%
5	9.558	963.27875	Peonidin3-O-(6’’’-feruloylsophoroside)-5-O-glucoside	4.0333%
6	10.578	1081.28479	Cyanidin 3-O-(6’’-caffeoyl-6’’’-p-coumarylsophoroside)-5-O-glucoside	3.6506%
7	9.72	773.19092	Delphinidin3-O-(6’’-p-coumaroylglucoside)-7-O-glucoside	1.5194%

MSn (*m*/*z*): MS fragmentation model; Rt (min): compound retention time (a) relative composition of anthocyanins calculated from peak areas recorded at 520 nm.

### 2.3 Animals, experimental design, and diets

The study involved the selection of thirty 3-month-old castrated male Hainan black goats, with an average weight of 11.30 ± 0.82 kg. A completely randomized block design was used to divide the goats into two groups, each comprising fifteen goats. The goats were divided into two groups: the control (CON) group, which received a basal diet, and the treatment (DAC) group, which received the basal diet supplemented with 40 mg/kg body weight (BW) of *Dioscorea alata L.* anthocyanins. Table 2 displays the composition and nutritional content of the basal diet. The entire experimental period was conducted over 67 days, which included a pre-trial phase lasting 7 days followed by a 60-day trial period. Each animal was housed individually in separate enclosures. During the adaptation period, the lambs were dewormed, ear-tagged, and vaccinated.

**Table 2:** Composition and nutritional level of the diets of Hainan black goats

Item	Concentrate	King grass
Raw materials %		
Corn	72.50	—
Soybean meal	10.00	—
DDGs	8.20	—
Shell powder	2.00	—
Baking soda	1.00	—
CaHPO₄	0.40	—
Caco_3_	0.40	—
Salt	0.50	—
Premix^1^	5.00	—
Total	100	—
Chemical composition^2^		
DM %	90.53	16.06
DE (MJ/kg)	12.97	—
CP %	12.48	6.40
EE %	3.63	2.17
Ash %	11.77	11.50
NDF %	59.88	68.87
ADF %	23.04	36.15
Ca %	0.53	0.23
P %	0.37	0.27

5% premix^1^: Vitamin A, 200,000 IU/kg; Vitamin D3, 60,000 IU/kg; Vitamin E, 550 IU/kg; D-Biotin, 0.3 mg/kg; Niacin, 350 mg/kg; Iron, 15 g/kg; Copper, 0.5 g/kg; Manganese, 2.4 g/kg; Zinc, 3 g/kg; Iodine, 20 mg/kg; Cobalt, Selenium, 10 mg/kg. All^2^ values, except for digestible energy, were measured experimentally.

To ensure accurate intake of DAC, a small quantity of pelletized feed was pulverized and thoroughly blended with DAC before being individually fed to each goat. Subsequently, the goats were provided with a combination of concentrate feed and king grass. Fresh king grass and concentrated feed were given twice daily, approximately at 8:00 am and 6:00 pm. Water and trace mineral salt blocks were provided *ad libitum* for consumption. The feed intake was periodically adjusted every 4 to 5 days, ensuring a residual feed of 5% to 10% remained. Measurements for dry matter intake (DMI) and feed residue were conducted on a daily basis, while weekly records of body weight were documented prior to each feeding. The calculation of average daily gain (ADG) involves dividing the weight gain by the specific experimental period for analysis. The determination of the feed conversion ratio (FCR) was conducted in a professional manner by dividing DMI by ADG.

### 2.4 Sample collection and processing

On both day 30 and day 60 of the trial period, blood samples were collected from both experimental groups. Prior to the morning feeding, a 10 mL blood collection was conducted from the jugular vein of each goat and transferred into a vacuum blood collection tube. The tubes were then promptly placed in a refrigerated centrifuge to facilitate plasma separation. The obtained plasma, used for further analysis of antioxidant and immune indicators, was stored at a temperature of −20°C in a freezer.

At the end of the feeding trial, a total of five lambs from each group, selected based on closely matching body weights (BW) with the group’s average BW, were chosen for slaughter to evaluate both carcass characteristics and meat quality. To determine the slaughter yield percentage, the goats’ final body weight (FBW) was recorded before slaughter. On the day of slaughter, the hair, organs, head, forelimb knee joints, and hindlimb toe joints were removed, and the carcass weight was recorded. Fresh meat samples (30 g) from the longissimus dorsi were collected from each lamb within 1 h after slaughter and placed in self-sealing bags. The meat samples were stored in the bags at a temperature of 4°C for further analysis of meat quality following standard protocols.

Following that, the carcasses were subjected to a 24-h chilling period at a temperature of 4°C. Subsequently, the half carcass located on the left side was then transferred to the meat quality laboratory. In order to evaluate the attributes of carcass quality, dissection was performed between the 12th and 13th ribs, which involved measuring the fat thickness at the 12th rib and the area of the longissimus dorsi (LM).

### 2.5 Analysis of carcass characteristics and meat quality

To assess the cross-sectional area of the latissimus dorsi (LD), we followed the evaluation procedure outlined by Wang et al. (20). Sulfur label paper was used to outline the cross-section in the intercostal region between the 12th and 13th ribs. The backfat thickness was assessed through caliper measurements, which were subsequently adjusted by considering the average backfat thickness between the 12th and 13th ribs. Muscle pH value was determined using a 1 cm-length glass electrode pH meter (HI99161, Hanna Instruments, Italy). The pH measurements were performed over a duration of 45 min and 24 h, and the mean of three readings was documented. Meat color attributes were assessed at three distinct sites using a spectrophotometer (Konica Minolta CR-400, Konica Minolta Camera Inc., Japan). To measure drip loss, the LD sample was suspended parallel to the orientation of the muscle fibers and placed in a sealed environment at 4°C for a period of 24 h. Following the removal of the sample from the bag, it was delicately wiped and subsequently reweighed. Cooking yield was measured by weighing trimmed LD (20–30 g), heating it in an 85°C water bath for 40 min, absorbing surface moisture with filter paper, and weighing it after cooling to constant temperature. Cooking yield was calculated by determining the proportion of the weight after cooking to the weight before cooking, expressed as a percentage. Shear force was evaluated according to the method of Destefanis (21).

### 2.6 Nutritional quality analysis of meat

The AOAC method was employed to analyze the routine nutrient quality components of the meat (22). The moisture content of Longissimus dorsi (LD) samples obtained from Hainan black goats was evaluated using a thorough drying process in an oven maintained at a constant temperature of 105°C. The calculation of crude protein content involved the application of the Kjeldahl method, specifically for nitrogen determination. The determination of EE content was conducted using the Soxhlet extraction method, following a similar protocol. The quantification of total ash levels was achieved through calcination of the sample in a crucible at a controlled temperature of 550 ± 25°C for 4 h, followed by calculation of the remaining inorganic matter.

### 2.7 Analysis of plasma antioxidant capacity and immune function

The quantification of SOD, GSH-Px, CAT, T-AOC, and MDA levels in plasma samples was performed using commercially available test kits from (Jiancheng Bioengineering Institute, Nanjing, China). The analysis was conducted in accordance with the provided guidelines.

Quantification of IgA, IgM, IgG, IL-1β, IL-6, IL-10, and TNF-α in plasma was performed using ELISA kits obtained from (Jiangsu Meibiao Biotechnology Co., Ltd. in Yancheng, China). All measurements were conducted in strict adherence to the recommended protocols.

### 2.8 Statistical analysis

The quantitative data acquired in this study were analyzed using SPSS software (Version 17.0, SPSS Inc., Chicago, United States). An independent-samples t-test was performed to assess significant differences in average values among the treatments. The outcomes consisted of mean values, corresponding *p*-values, and the standard error of the mean (SEM). GraphPad Prism 8.0 software (GraphPad Software Inc., La Jolla, United States) was employed to present the statistical findings visually. In this study, statistical significance was defined as *p* < 0.05, while high statistical significance was considered as *p* < 0.01.

## 3 Results

### 3.1 Growth performance

The DAC group did not exhibit any statistically significant effects (*p* > 0.05, Table 3) on the final weight, ADG, DMI, and FCR of Hainan black goats at different stages, when compared to the CON group.

**Table 3:** Effect of dietary supplementation of DAC on growth performance of Hainan black goat.

Items	Treatments		SEM	*p*-value
	CON	DAC		
0-30d				
IBW	11.29	11.32	0.20	0.94
BW	13.3	12.98	0.31	0.61
DMI^1^ (g/d)	414.42	405.28	5.31	0.45
DMI^2^ (g/d)	112.24	102.43	12.83	0.75
ADG (g/d)	66.76	57.22	4.88	0.39
FCR	7.89	8.37	0.65	0.62
31-60d				
FBW	15.43	15.46	0.47	0.97
DMI^1^ (g/d)	528.51	539.19	27.55	0.87
DMI^2^ (g/d)	109.45	112.32	8.71	0.89
ADG (g/d)	78.89	84.26	10.91	0.84
FCR	8.04	7.72	0.53	0.87
0-60d				
DMI^1^ (g/d)	471.47	472.23	13.19	0.98
DMI^2^ (g/d)	110.85	107.34	8.72	0.87
ADG (g/d)	69.12	70.74	3.23	0.84
FCR	8.42	8.20	0.13	0.45

SEM = standard error; DMI^1^ = (concentrated feed) dry matter intake; DMI^2^ = (King grass) dry matter intake; IBW = initial body weight; FBW = final body weight; ADG = average daily gain; FCR= feed conversion ratio.

### 3.2 Carcass characteristics and meat quality

No statistically significant differences (*p* > 0.05, Table 4) were observed between the various treatments in terms of backfat thickness, carcass weight, slaughter yield, and the longest muscle area of the back. No significant differences were found between the two groups in terms of the L*, a*, and b* values in the LM (*p* > 0.05, Table 5). The addition of DAC to the diet significantly improved the pH value and cooking yield of the LM after 24 h, as compared to the CON group (*p* < 0.05, Table 5). Furthermore, the DAC group showed a noteworthy reduction in shear force of the LM compared to the CON group (*p* < 0.05, Table 5).

**Table 4:** Effect of dietary supplementation of DAC on carcass characteristics of Hainan black goat

Items	Treatments		SEM	*p*-value
	CON	DAC		
Carcass weight (kg)	7.67	7.52	0.14	0.62
Dressing percentage %	46.62	47.75	0.98	0.59
Back fat thickness (mm)	6.86	6.47	0.72	0.81
LM area (cm^2^)	12.43	13.07	1.04	0.77

SEM = standard error; LM = longissimus dorsi muscle.

**Table 5:** Effect of dietary supplementation of DAC on meat quality of Hainan black goat.

Items	Treatments		SEM	*p*-value
	CON	DAC		
L*	36.68	36.64	0.28	0.95
a*	13.64	14.21	0.19	0.13
b*	8.68	8.58	0.23	0.84
PH_45min_	6.84	6.78	0.04	0.49
PH_24h_	6.08^a^	6.21^b^	0.03	0.04
Drip loss %	3.96	3.48	0.29	0.06
Cooking yield %	55.71^a^	58.49^b^	0.69	0.04
Shear force N	85.86^a^	76.02^b^	2.22	0.02

SEM = standard error; color measurements: L* = lightness; a* = red; b* = yellow; a, b Means followed by different superscripts are significantly different (*p* < 0.05).

### 3.3 Nutritional quality of meat

No significant disparities were found in the moisture and ash content of the longissimus muscle (LM) between the Hainan black goats in the DAC and CON groups (*p* > 0.05, Table 6). However, it should be noted that the fat and protein content of the DAC group was slightly higher compared to the CON group, although the difference was not statistically significant (*p* > 0.05, as shown in Table 6).

**Table 6:** Effect of dietary supplementation of DAC on the nutritional quality of the longissimus dorsi muscle of Hainan black goat.

Items	Treatments		SEM	*p*-value
	CON	DAC		
Moisture (%)	75.60	75.48	0.18	0.75
Protein (%)	22.81	22.89	0.15	0.80
Fat (%)	2.37	2.60	0.15	0.45
Ash (%)	1.16	1.03	0.02	0.31

SEM = standard error.

### 3.4 Plasma antioxidant index

**Figure 1:**
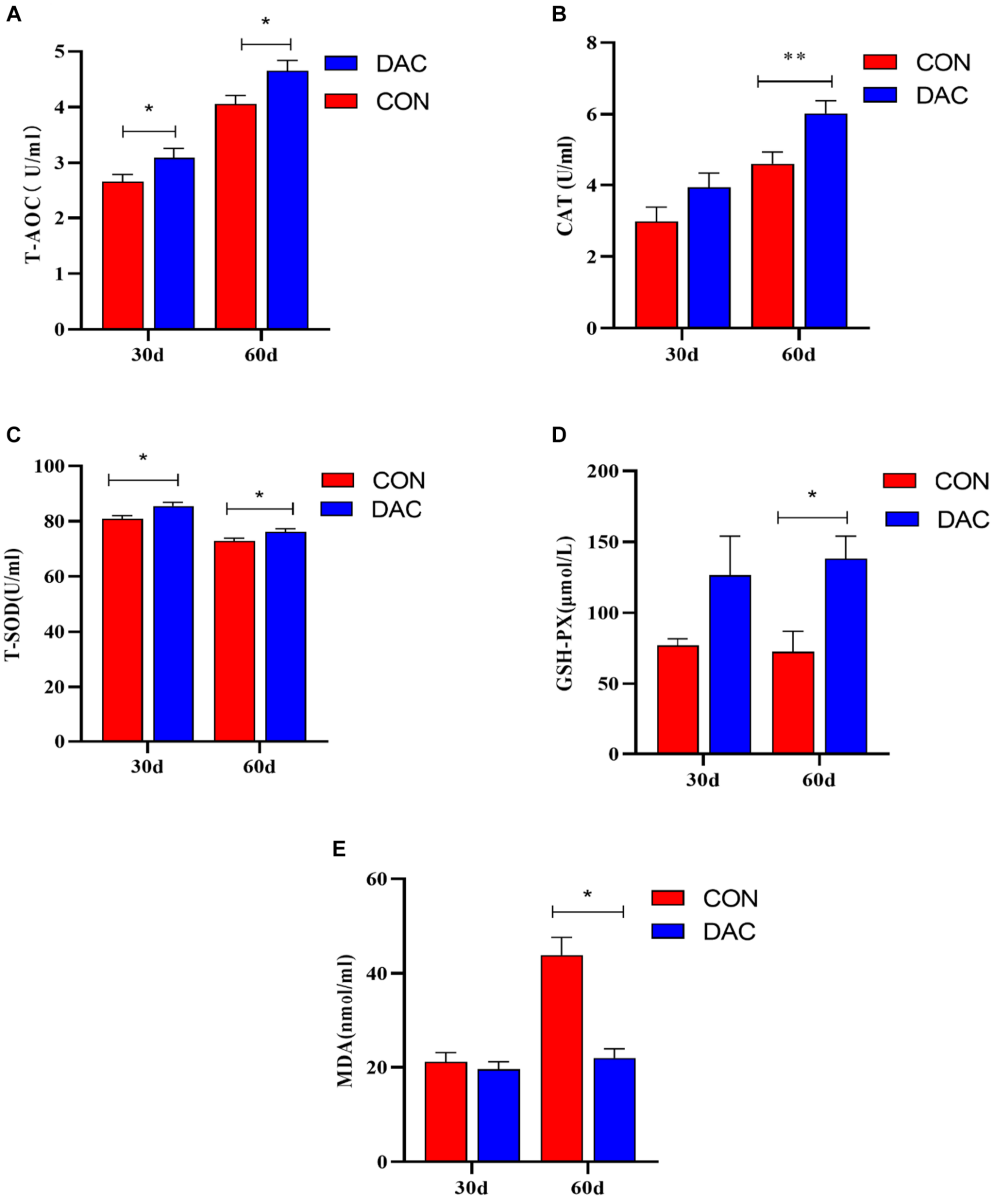
Effect of dietary supplementation with DAC on plasma antioxidant indexes in Hainan black goats. The primary indicators reflecting antioxidant capacity include: **(A)** T-AOC = total antioxidant capacity; **(B)** CAT = catalase; **(C)** T-SOD = total superoxide dismutase; **(D)** GSH-Px = glutathione peroxidase; and **(E)** MDA = malondialdehyde; results are expressed as means ± SEM. * indicated significant difference (*p* < 0.05), and ** indicated extremely significant difference (*p* < 0.01).

Figure 1 illustrates the impact of DAC dietary supplementation on antioxidant indicators within the plasma of Hainan black goats. After 30 days, the addition of DAC to the diet led to a noteworthy enhancement in both plasma T-AOC and T-SOD concentrations, with statistical significance (*p* < 0.05). After 60 days of incorporating DAC into the diet, the group supplemented with DAC displayed substantial improvements in plasma T-AOC, T-SOD, and GSH-Px concentrations compared to the CON group, with statistical significance (*p* < 0.05). Moreover, the DAC group also experienced a noteworthy elevation in plasma CAT concentration (*p* < 0.01). Additionally, there was a significant decrease in MDA content observed in the DAC group when compared to the CON group, with statistical significance (*p* < 0.01).

### 3.5 Plasma immune parameters

**Figure 2:**
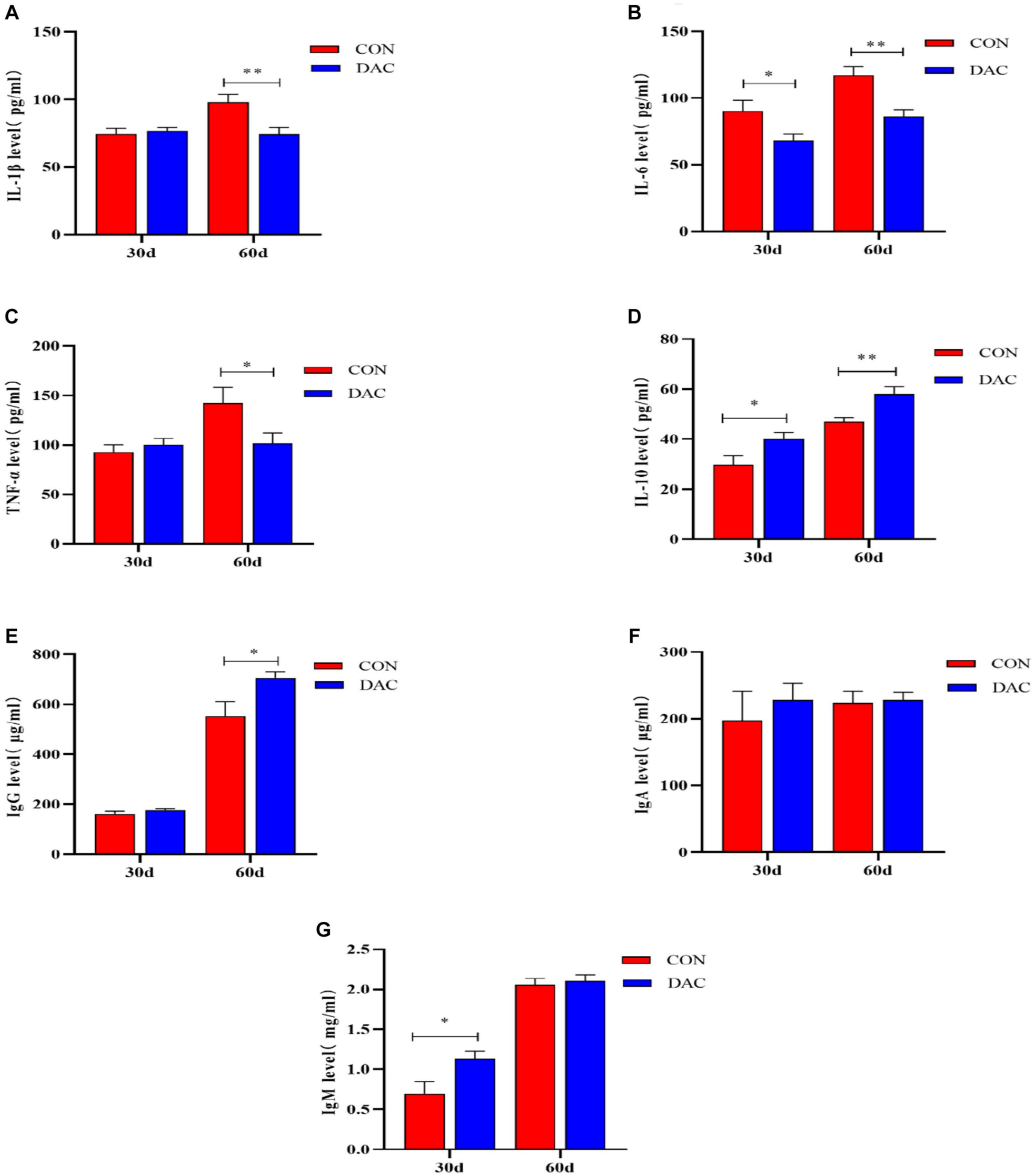
Effect of dietary supplementation with DAC on Plasma Immune Parameters in Hainan black goats. The primary indicators reflecting immune function include: **(A)** IL-1β = Interleukin-1β; **(B)** IL-6 = Interleukin-6; **(C)** TNF-α = tumor necrosis factor-α; **(D)** IL-10 = Interleukin-10; **(E)** IgG = Immunoglobulin G; **(F)** IgA = Immunoglobulin A; and **(G)** IgM = Immunoglobulin M; Results are expressed as means ± SEM. * indicated significant difference (*p* < 0.05), and ** indicated extremely significant difference (*p* < 0.01).

The immune parameters among different treatment groups are presented in Figure 2. After a 30-day intervention of DAC dietary supplementation, the DAC group demonstrated a significant decrease in IL-6 levels in comparison to the CON group, with statistical significance (*p* < 0.05). Additionally, DAC dietary supplementation led to noteworthy increases in plasma IL-10 and IgM levels (*p* < 0.05). In a comprehensive 60-day experimental trial on Hainan black goats, the incorporation of DAC into their diet resulted in a remarkable reduction in plasma levels of IL-1β (*p* < 0.01), IL-6 (*p* < 0.01), and TNF-α (*p* < 0.05). Additionally, the dietary supplementation of DAC in Hainan black goats significantly elevated plasma levels of IgG (*p* < 0.05) and IL-10 (*p* < 0.01).

## 4 Discussion

The growth performance of animals is easily influenced by factors such as breed, feed composition, and environmental conditions (23–25). Anthocyanins, belonging to the flavonoid group, are commonly applied to animal production as feed additives. Plant-derived flavonoids, which are categorized as secondary metabolites, have been previously shown to play a vital role in the growth of ruminant animals (26). However, the reports on the effects of anthocyanin-rich black sugarcane and red corn on goat growth performance and carcass characteristics did not show any significant changes (9, 27). Consistent with previous research findings, the inclusion of DAC in the diet did not have a significant impact on the growth performance and carcass characteristics of Hainan black goats in this study. Nevertheless, other studies have reported favorable outcomes (28). The potential factors contributing to the observed differences in outcomes may include variations in the formulation of anthocyanin extract, slaughter age, and differences in the bioavailability of phenolic compounds in ruminant and monogastric animals (29). Based on the findings of this study, it can be deduced that the inclusion of DAC in the animal diet does not adversely impact animal performance. These findings can provide valuable insights for future applications in goat production.

Meat quality can be effectively assessed by considering key indicators such as pH, cooking yield, and shear force measurements. The degree of decrease in muscle pH may be associated with the glycogen content in skeletal muscles (30). Glycogen is converted to lactic acid through anaerobic or glycolytic pathways, resulting in the production of H+ and subsequently decreasing muscle pH (31). However, the concentration of glycogen is influenced by various factors, including pre-slaughter stress (32). Jiao et al. reported that the addition of grape seeds rich in anthocyanins to sweet sorghum could increase the pH value of lamb meat 24 h after slaughter (33). In this study, it was found that the addition of DAC to the diet can increase the pH value of Hainan Black Goat muscles 24 h after slaughter. The observed phenomenon is likely attributed to the relief of pre-slaughter stress in lambs, which subsequently affects muscle glycogen storage and inhibits muscle glycolysis. Previous research has reported that meat with higher cooking yields tends to lose less moisture during the heating process (34). Our research findings demonstrate that the DAC group exhibits higher cooking yield, indicating the potential of anthocyanins in enhancing muscle water-holding capacity and juiciness. Shear force serves as the most direct and indicative measure of meat tenderness (35). Conversely, shear force is influenced by factors such as collagen content, characteristics of collagen proteins, and muscle fiber structure in connective tissues (36). Moreover, the tenderness of meat is also associated with the speed of glycolysis and the ultimate pH level (37). Scientific evidence supports the claim that polyphenols can enhance tenderness in meat by suppressing the expression of fiber-related genes involved in glycolysis, thereby reducing shear force (38). In this study, the incorporation of DAC in the diet resulted in a reduction in shear force measurement. Furthermore, in combination with our pH value results, this suggests that DAC may influence glycolysis, thereby impacting muscle fiber structure, leading to a decrease in shear force and an improvement in tenderness. Similar results have been observed in ruminant animals with the dietary inclusion of plant extracts rich in anthocyanins (39, 40).

Meat consists of water, proteins, lipids, minerals, and a small amount of carbohydrates. No significant variations were detected in the nutritional composition across the groups investigated, suggesting that the inclusion of DAC did not impact the regulation of nutritional components in goats. Our findings are consistent with the results reported by Salzano et al. (41), which demonstrated that feeding goats with anthocyanin-rich extracts from red oranges and lemons did not result in any differences in the general nutrition facts of the meat. The limited absorption of anthocyanins in animals may explain this phenomenon, although further research is necessary to confirm this hypothesis.

The proper functioning of the body and its organs depends on the presence of free radicals (FR), ROS, and reactive nitrogen species (RNS), as well as a well-balanced correlation between these free radicals and the antioxidant system in healthy animals (42). Under normal circumstances, the body can improve animal health by eliminating excessive free radicals through endogenous antioxidant enzymes such as SOD, GPX, and CAT (43). However, the body may occasionally experience oxidative stress (OS) due to an imbalance in this state. It is typically necessary to supplement natural antioxidants in livestock diets to alleviate oxidative stress in animals. Anthocyanins, as natural antioxidants, possess the ability to inhibit or prevent compound oxidation by efficiently scavenging FR and reducing OS (44). It has been confirmed that anthocyanins are absorbed into the bloodstream upon consumption (45). In our study, feeding with DAC for 30 and 60 days significantly increased the activity of SOD and T-AOC in plasma. This outcome can possibly be attributed to the absorption of anthocyanins into the bloodstream, which alleviates OS due to the hydrogenating (electron-donating) ability of flavonoid molecules. Furthermore, feeding with DAC for 60 days resulted in higher activity of CAT and GPX, as well as significantly lower activity of malondialdehyde (MDA). Our findings corroborate with the results obtained by Taethaisong et al. (46) who fed goats with diets containing purple taro leaves rich in anthocyanins. They observed an increase in plasma levels of T-AOC, SOD, GPX, and CAT, along with a significant decrease in MDA concentration. It has been reported that the expression levels of SOD, GPX, and CAT are primarily regulated by nuclear factor-E2-related factor 2 (Nrf2), which is a key factor in protecting against oxidative stress (47). In addition, the consumption of silage feed made from purple corn stalks rich in anthocyanins was found to increase the levels of SOD in plasma and the expression levels of SOD2, GPX1, and GPX2 mRNA in the mammary glands (48). Therefore, it is hypothesized that dietary supplementation of DAC to increase antioxidant enzyme activity may be associated with the enhancement of the body’s antioxidant capacity through the modulation of the Nrf2 signaling pathway by anthocyanins (49). However, further research is needed to elucidate the exact reasons and mechanisms underlying this hypothesis.

The immune function of ruminant animals is crucial for maintaining their overall health status. Immunoglobulins play a significant role in host-mediated humoral immunity, where IgG activates the complement system and resists the invasion of various bacteria and toxins (50, 51). IgM binds to complement and dissolves pathogens (52). Current research indicates that the DAC group exhibited increased concentrations of IgM and IgG in the plasma at 30 days and 60 days, respectively. A study reported that supplementing the diet with hibiscus anthocyanins enhanced the immunity level of IgG in the spleens of chickens (53). The enhanced immune globulin (IgM and IgG) activities demonstrated by the DAC group may be attributed to the antioxidant and antibacterial capacities of the extract, primarily driven by its flavonoid content (mainly anthocyanins) (54, 55). Oxidative stress can induce the activation of nuclear factor-kappa B (NF-κB) (56), which in turn triggers inflammatory responses (57). The activation of NF-κB has been shown to promote gene expression and the synthesis of various pro-inflammatory cytokines (58). Cytokines serve as crucial indicators of the inflammatory status within organisms, which can be classified into anti-inflammatory interleukin-10 (IL-10) and pro-inflammatory cytokines including interleukin-1β (IL-1β), interleukin-6 (IL-6), and tumor necrosis factor-α (TNF-α), among others (59). Based on a study, it has been verified that anthocyanin supplements efficiently inhibit the transactivation of NF-κB, which leads to a reduction in the plasma concentrations of pro-inflammatory chemokines, cytokines, and mediators (60). Our research findings demonstrate that dietary supplementation with DAC can reduce the levels of pro-inflammatory cytokines (IL-1β, IL-6, TNF-α) in plasma, while promoting an increase in the concentration of anti-inflammatory cytokine IL-10. This effect becomes more pronounced after feeding with DAC for 60 days. The supplementary inclusion of Dioscorea in the diet of goats exhibited a similar trend to our findings in terms of plasma pro-inflammatory factors (61). The study findings suggest that DAC supplementation in the diet of weaned Hainan black goat lambs effectively mitigates serum inflammatory response. This process involves the stimulation of anti-inflammatory factors and suppression of pro-inflammatory factors, thereby boosting their immune function.

## 5 Conclusion

Based on the present results, it can be concluded that the inclusion of DAC in the diet does not affect the growth performance and carcass characteristics of Hainan black goats. However, it does improve meat quality to some extent. The addition of 40 mg/kg BW of DAC to the diet leads to an improved immune response and antioxidant capacity through increased levels of IgM, IgG, IL-10, GSH-Px, SOD, CAT, and T-AOC. The potential of *Dioscorea alata L.* anthocyanins in improving the meat quality, antioxidant capacity, and immune function of Hainan black goats signifies their promising role as feed additives in goat production systems. Further research is needed to elucidate the mechanisms of action and determine the ideal dosage and duration of supplementation with anthocyanins derived from *Dioscorea alata L.*

## Data availability statement

The original contributions presented in the study are included in the article/supplementary material, further inquiries can be directed to the corresponding author.

## Ethics statement

The animal study was approved by the Ethics Committee of Hainan University (Haikou, China). The study was conducted in accordance with the local legislation and institutional requirements.

## Author contributions

HF: Conceptualization, Methodology, Writing – original draft. HS: Conceptualization, Funding acquisition, Methodology, Visualization, Writing – review & editing. FY: Formal analysis, Investigation, Validation, Writing – review & editing. YY: Data curation, Project administration, Software, Writing – review & editing. XW: Conceptualization, Methodology, Resources, Supervision, Writing – review & editing.
